# Realistic numerical simulations of diffusion tensor cardiovascular magnetic resonance: The effects of perfusion and membrane permeability

**DOI:** 10.1002/mrm.29737

**Published:** 2023-07-06

**Authors:** Ignasi Alemany, Jan N. Rose, Pedro F. Ferreira, Dudley J. Pennell, Sonia Nielles‐Vallespin, Andrew D. Scott, Denis J. Doorly

**Affiliations:** ^1^ Department of Aeronautics Imperial College London London UK; ^2^ Cardiovascular Magnetic Resonance Unit, Royal Brompton Hospital Guy's and St Thomas' NHS Foundation Trust London UK; ^3^ National Heart and Lung Institute Imperial College London London UK

**Keywords:** DT‐CMR, modeling, Monte Carlo, perfusion

## Abstract

**Purpose:**

To study the sensitivity of diffusion tensor cardiovascular magnetic resonance (DT‐CMR) to microvascular perfusion and changes in cell permeability.

**Methods:**

Monte Carlo (MC) random walk simulations in the myocardium have been performed to simulate self‐diffusion of water molecules in histology‐based media with varying extracellular volume fraction (ECV) and permeable membranes. The effect of microvascular perfusion on simulations of the DT‐CMR signal has been incorporated by adding the contribution of particles traveling through an anisotropic capillary network to the diffusion signal. The simulations have been performed considering three pulse sequences with clinical gradient strengths: monopolar stimulated echo acquisition mode (STEAM), monopolar pulsed‐gradient spin echo (PGSE), and second‐order motion‐compensated spin echo (MCSE).

**Results:**

Reducing ECV intensifies the diffusion restriction and incorporating membrane permeability reduces the anisotropy of the diffusion tensor. Widening the intercapillary velocity distribution results in increased measured diffusion along the cardiomyocytes long axis when the capillary networks are anisotropic. Perfusion amplifies the mean diffusivity for STEAM while the opposite is observed for short diffusion encoding time sequences (PGSE and MCSE).

**Conclusion:**

The effect of perfusion on the measured diffusion tensor is reduced using an increased reference *b*‐value. Our results pave the way for characterization of the response of DT‐CMR to microstructural changes underlying cardiac pathology and highlight the higher sensitivity of STEAM to permeability and microvascular circulation due to its longer diffusion encoding time.

## 1 INTRODUCTION

Diffusion Tensor Imaging is an in‐vivo non‐invasive MR technique for characterizing the underlying tissue microstructure. This MR technique measures the self‐diffusion of water molecules within a voxel providing information on the magnitude of diffusion, the degree of diffusion anisotropy and its orientation.^1^, ^2^ Diffusion tensor imaging applied to the heart presents several challenges such as the intrinsic deformation during the cardiac cycle or the respiratory associated movement.^3^, ^4^ However, recent developments^4^, ^5^ in diffusion tensor cardiovascular magnetic resonance (DT‐CMR) have overcome these difficulties providing in‐vivo insights into the microstructural abnormalities underlying various myocardial pathologies.^6^, ^7^ Typical DT‐CMR sequences allow water molecules to diffuse tens of micrometers probing the local tissue microstructure. However, the MR signal is measured in a much larger voxel region ($3\times 3\times 8\;{\text{mm}}^{3}$) effectively averaging the measured tissue microstructure. Furthermore, the link between a given change in microstructure and the corresponding change in DT‐CMR parameters is not well established. The comprehension of this missing link between the MR image data and the microscopic structure lends itself to investigations using Monte Carlo (MC) random walk simulations^8^, ^9^, ^10^ or finite element method (FEM) based simulators,^11^, ^12^ which have the power to elucidate the sensitivity of the DT‐CMR signal to confounding factors or biological parameters present in the tissue microstructure.

The first cardiac microstructure numerical phantom^8^, ^9^ utilized MC simulations and simplified cardiomyocytes to cylindrical geometries with differing hexagonal cross sections and lengths. Recently,^10^ we generated a more realistic numerical phantom based on manually segmented histology. In this virtual tissue, the membranes were considered impermeable and radially adjacent blocks were rotated in the local coordinate system to simulate the helix angle (HA) present in the myocardium.^13^ By performing MC random walk simulations, we showed that the distribution of extracellular space (ECS) has a measurable impact on the DT‐CMR parameters. We also showed that, in the case of permeable membranes, the distances diffused in long diffusion time sequences such as Stimulated Echo Acquisition Mode (STEAM) would be sufficient to span multiple cardiomyocytes. Simultaneously, experimental studies have shown that cardiomyocytes are not fully impermeable and exchange rates for intra‐extracellular water exchange have been reported from 6 to 30 Hz.^14^, ^15^, ^16^ Mathematical models^17^, ^18^ can be utilized in the MC random walk simulations to accurately represent the cell permeability by solving the interface boundary condition.^19^ However, these models suffer from numerical limitations^20^ when the membrane separates media with different diffusivites.

DT‐CMR techniques are not only sensitive to molecular diffusion but also to the blood flow in capillaries.^21^ Numerical simulations^22^, ^23^ have been performed in the brain where the capillary network is modeled as isotropic and several theories have been proposed for characterizing the microcirculation in the capillary network.^21^ Due to the assumption that the capillaries are randomly oriented, most of these models do not translate well to cardiac and skeletal muscle tissue as the capillary network is anisotropic and, on average, parallel to the myocytes.^24^, ^25^ More recent numerical simulations^26^, ^27^, ^28^ have created anisotropic capillary network models to simulate the effect of several perfusion parameters on the diffusion weighted MR signal.

The aim of this work is to study the sensitivity of DT‐CMR parameters to changes in cell membrane permeability and microvascular perfusion. The simulations of the diffusion process use a new permeability model^20^ that improves upon the treatment of the interface boundary condition^19^ between regions of different diffusivity as compared to previous numerical simulations of diffusion tensor imaging. In a similar manner, the microvascular perfusion signal has been modeled assuming that a group of particles travel at constant velocities through a capillary network anisotropically oriented according to a Dimroth–Watson distribution.^29^, ^30^ The sensitivity of the tensor to perfusion has been studied for typical DT–CMR sequences by adding a weighted contribution of the perfusion signal to the simulated diffusion signal, providing a more complete MC random walk simulation than in previous work.

## 2 METHODS

Here we introduce the histology‐based substrate, the treatment of the interface boundary condition substrate, the permeability model and the key elements of the MC random walk simulations. Next, we present the perfusion model and its incorporation into our DT‐CMR simulations.

### 2.1 Histology‐based substrate

**FIGURE 1:**
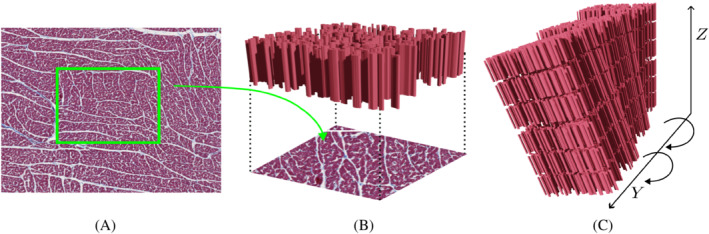
(A) Illustration of the two‐dimensional histology‐image and the region of interest (ROI) that was manually segmented. (B) Extrusion of the ROI in the long axis of the cardiomyocytes recreating a block of tissue of $500\times 400\times 120\;\mu m^{3}$. (C) Representation of the rotation applied to adjacent blocks in order to simulate the transmural variation in the helix angle (HA). Note that for illustration purposes, the three‐dimensional model contains few block repetitions with fewer cardiomyocytes than the actual histology‐image and the spacing between the blocks is not realistic.

The histology‐based substrate is constructed by replicating a block of tissue of $495\times 392\times 127\;\mu m^{3}$.^10^ This repeating block of tissue is generated by extruding 1182 contours obtained from manual segmentation of porcine histology (Masson staining).^31^ The cardiomyocyte cross‐sections were segmented from a region of interest in the mesocardium where the imaging plane was approximately perpendicular to the cardiomyocyte long axis. As observed in Figure 1, each block of tissue is rotated around the myocardial radial direction (Y‐direction in Figure 1) by applying a rotation of $10^{\circ }\;{\text{mm}}^{-1}$ to account for the transmural HA variation observed in the myocardium.^4^, ^13^, ^32^ The different blocks are also shifted half a block every other row in the long axis of the cardiomyocytes (Z‐direction in Figure 1) to eliminate long straight channels.

### 2.2 MC random walk

The Brownian motion or self‐diffusion of water molecules can be modeled as a MC random walk of massless, noninteracting particles. Particles are uniformly seeded in the histology‐based substrate inside a cuboid representing the dimensions of a typical DT‐CMR voxel ($2800\times 2800\times 8000\;\mu m^{3}$) and a buffer zone to minimize biases due to particles entering/leaving the voxel during the simulation.^10^ Note that a typical DT‐CMR voxel has a larger slice thickness (Z‐direction) than the in‐plane resolution to maintain an acceptable signal‐to‐noise ratio during the short acquisition times. However, additional simulations have shown similar tensor results for an isotropic voxel (refer to Table S1). At each time step δt, the position of each particle $\vec{X}(t)$ is updated using a random constant step‐size vector $\vec{R}$, such that for each component $R_{i}$, there is the same equal probability of moving one unit in either possible direction ($R_{i}=+1$ or $R_{i}=-1$). After one whole step,

(1) $$\vec{X}(t+\delta t)=\vec{X}(t)+\sqrt{2D\delta t}\vec{R},$$

where $\vec{R}$ is scaled by the local diffusivity $\sqrt{2D\delta t}$ which can either be intracellular, $D_{\text{ICS}}$, or extracellular, $D_{\text{ECS}}$. The intersections are resolved using additional substeps and the transit model described in Section 2.3. The cumulative phase for each particle is defined by

(2) $$\phi_{i}=\gamma \int_{0}^{T}\vec{X}(t)\cdot \vec{G}(t)\;dt,$$

where $\vec{G}(t)$ is the time‐dependent gradient vector. At the end of the random walk, the diffusion signal attenuation is described by the following equation

(3) $$\frac{S_{\text{diff}}(b,\vec{e_{g}})}{S_{0}}=\frac{1}{N_{p,\text{diff}}}|\overset{N_{p,\text{diff}}}{\underset{i=1}{\sum }}e^{-j\phi_{i}}|,$$

where $S_{\text{diff}}/S_{0}$ is the normalized diffusion signal, $\vec{e_{g}}$ the gradient direction, b a specific sequence *b*‐value^33^ and $N_{p,\text{diff}}$ the number of particles utilized in the diffusion MC random walk simulation. To validate our in‐house MC random walk algorithm we compared to a finite element (FE) solution in a representative two‐dimensional geometry (refer to Figures S1 and S2).

### 2.3 Permeability

In this work we assume uniform sarcolemma permeability $\kappa_{\text{sarco}}$ for all cardiomyocytes. A range of $\kappa_{\text{sarco}}$ values is considered, to study the effect of intra‐extracellular water exchange on DT‐CMR parameters, from impermeable $\kappa_{\text{sarco}}=0\mu m\;{\text{ms}}^{-1}$ to highly permeable $\kappa_{\text{sarco}}=0.05\;\mu m\;{\text{ms}}^{-1}$.^14^, ^15^, ^16^ Whenever a particle interacts with a cell membrane, a probability of transit $p_{t}$ is computed via a transit model that represents the permeability embedded in the membrane boundary condition.^19^, ^20^ This transit model, that we refer to as the hybrid model,^20^ treats the membrane permeability and the step change in diffusion as successive barrier interactions. Doing so reduces the complexity of the transit probabilities to

(4) $$p_{t_{\text{ECS}\to \text{ICS}}}=p_{b}\cdot p_{d}p_{t_{\text{ICS}\to \text{ECS}}}=p_{b},$$

respectively, for transit from ECS to ICS and viceversa, where $p_{b}$ is the membrane transit probability and $p_{d}$ the probability of a particle transitioning between two media of different diffusivities. The membrane transit probability $p_{b}$ is derived by Fieremans et al.^17^ and the probability of transitioning between two media $p_{d}$ of different diffusivity is computed through a step‐balanced interface permeability.^34^ These two probabilities are given by the following equations

(5) $$p_{b}=\frac{2\kappa_{\text{sarco}}\delta x}{D_{\text{ICS}}+2\kappa_{\text{sarco}}\delta x}p_{d}=\sqrt{\frac{D_{\text{ICS}}}{D_{\text{ECS}}}},$$

where δx is the distance at the beginning of the step between the particle and the membrane surface projected onto the vector normal to the surface.

### 2.4 Diffusion experiments

We considered three different sequences; the Stejskal–Tanner monopolar pulsed‐gradient spin echo (PGSE),^35^ gradient duration modulated second‐order motion‐compensated spin echo (MCSE)^5^, ^36^ and monopolar STEAM.^3^, ^37^ Diffusion simulations were performed for each sequence considering two $G_{\max}$ values in order to match a reference *b*‐value of $b_{\text{ref}}=0.15\;\text{ms}\;\mu m^{-2}$ and a *b*‐value of $b=0.6\;\text{ms}\;\mu m^{-2}$. Sequence parameters are summarized in the Table S2. For each experiment we performed six simulations with the following constant parameters: $D_{\text{ECS}}=2.5\;\mu m^{2}\;{\text{ms}}^{-1}$, $D_{\text{ICS}}=1\;\mu m^{2}\;{\text{ms}}^{-1}$, $N_{t}=10^{4}$ (number of time steps) and $N_{p,\text{diff}}=10^{5}$ (number of particles) using MATLAB (R2017b) with the Parallel Computing Toolbox on the Imperial College High Performance Computing facility (utilizing up to 120 computing nodes and 240 GB of RAM).

### 2.5 Extracellular volume fraction

The effect of changes in extracellular volume fraction (ECV) is studied by shrinking/growing the manually segmented cardiomyocytes.^10^ The cardiomyocytes have been morphed obtaining a default healthy value of 24.69% and two extreme ECV values of 41.82% and 18.8% (see Figure S3).

### 2.6 Intercalated disks

**FIGURE 2:**
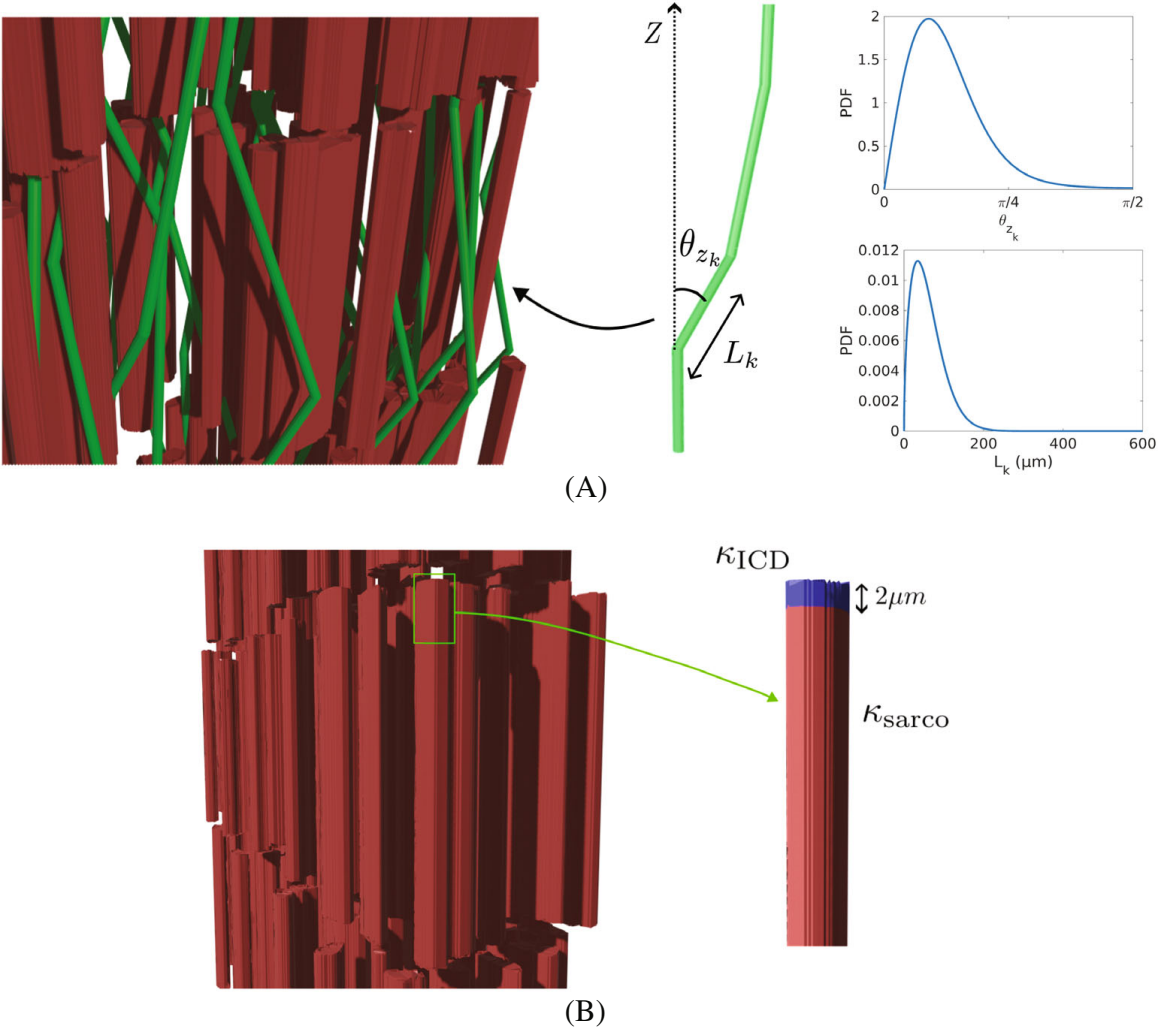
(A) 3D illustration of the capillary network with representative expanded (spacing increased for visualization) cardiomyocytes, showing a Dimroth–Watson distribution (upper PDF) for the zenith angle $\theta_{z_{k}}$ and Weibull distribution (lower PDF) for the length $L_{k}$ of the capillary segments. The PDF has been calculated considering parameters that are representative of the capillary network in the myocardium. (K=3.25 and μ=60μm, σ=40μm for the length distribution^24^). (B) 3D illustration of the intercalated disk (ICD) structures between the neighboring cardiomyocytes. A more detailed illustration for the two different permeabilities ($\kappa_{\text{sarco}}$ and $\kappa_{\text{ICD}}$) is depicted.

Intercalated disks (ICD) are complex structures connecting the ends of neighboring cardiomyocytes and are composed of three major complexes: desmosomes, fascia adherens and gap junctions.^38^ Desmosomes and fascia adherens reinforce the cardiomyocyte junction while gap junctions ensure the passage of electrical current between neighboring cardiomyocytes through intercellular channels.^39^ To study the sensitivity of the measured diffusion tensor to possible water restriction in the ICDs, we compare simulations for ICDs having the same permeability as the rest of the sarcolemma, with simulations for lower ICD permeability of $\kappa_{\text{ICD}}=0.005\;\mu {\text{m ms}}^{-1}$ ^40^ on the end caps of each cardiomyocyte. As depicted in Figure 2, this low permeability region has been extended 2μm along the long axis of the cardiomyocyte to ensure that the entire end cap is within the region of low permeability irrespective of any geometry irregularity.

### 2.7 Perfusion model

The microvascular circulation during and between diffusion encoding gradient waveforms causes alterations to the diffusion MR signal magnitude. The contribution of microvascular perfusion to the MR signal is investigated independently from diffusion simulations by simulating a group of particles following specific paths determined by an anisotropic model. As depicted in Figure 2, each particle travels at constant velocity v through different multiple straight segments traversing k segments, each with a length $l_{k}$ and orientation ${\vec{e}}_{x_{k}}$. The multiple segments are derived from an anisotropic model that simulates the orientation of the capillary network providing a simplified representation of the capillary orientation without the need for a detailed network. Note that the mean capillary orientation is always aligned with the mean orientation of the cardiomyocytes, thereby following the same HA rotation described in Section 2.1.

Similarly to random walk, the phase acquired by any particle is given by equation (2). For constant intercapillary velocity, the perfusion phase $\phi_{\text{perfusion}}$ can be simplified by summing the accumulated phase of each traversed segment. Following this simplification, $\phi_{\text{perfusion}}$ is normalized by the b‐value, gradient strength $G_{\max}$ and velocity v ^27^, ^41^ and is then only dependent on the orientation of the segments ${\vec{e}}_{x_{k}}$ and the gradient profile $\vec{G}(t)$ normalized in amplitude and time h(t/T), that is, $\vec{G}(t)=G_{max}h(t/T)\;\vec{e_{g}}$

(6) $$\Phi_{\text{perfusion}}=\frac{\phi_{\text{perfusion}}}{v\sqrt{bT}}=\frac{1}{a}\overset{N}{\underset{k=1}{\sum }}\vec{e_{g}}\cdot {\vec{e}}_{x_{k}}\int_{s_{k-1}}^{s_{k}}m_{0}(s)\;ds.$$

In the above $s_{k-1}$ and $s_{k}$ are the normalized times when the particle starts and completes a certain capillary segment, $m_{0}=\int_{0}^{s}h(s^{\prime })\;ds^{\prime }$ the normalized 0‐th gradient moment and a the normalized b‐value $a=\sqrt{b}/(\gamma G_{\max}T^{3/2})$. At the end of the simulation, the normalized perfusion signal $S_{\text{perfusion}}(b,\vec{e_{g}})/S_{0}$ is determined through Equation (3). The total normalized signal ($S(b,\vec{e_{g}})/S_{0}$) is defined by the weighted contribution:

(7) $$\frac{S(b,\vec{e_{g}})}{S_{0}}=f\frac{S_{\text{perfusion}}(b,\vec{e_{g}})}{S_{0}}+(1-f)\frac{S_{\text{diffusion}}(b,\vec{e_{g}})}{S_{0}},$$

where $\vec{e_{g}}$ is the gradient direction and f the perfusion fraction (i.e., the proportion of the detectable spins within the capillary network). The total signal loss is defined as $1-S(b,\vec{e_{g}})/S_{0}$ and has been obtained using a healthy ECV of 24.7% for the diffusion simulations with $\kappa_{\text{sarco}}=\kappa_{\text{ICD}}=0.03\;\mu m\;{\text{ms}}^{-1}$ and a default perfusion fraction of f=10% as echocardiographic studies^42^ report a volume fraction of blood in myocardium of 12.6%±1.8% for healthy individuals at rest.

**FIGURE 3:**
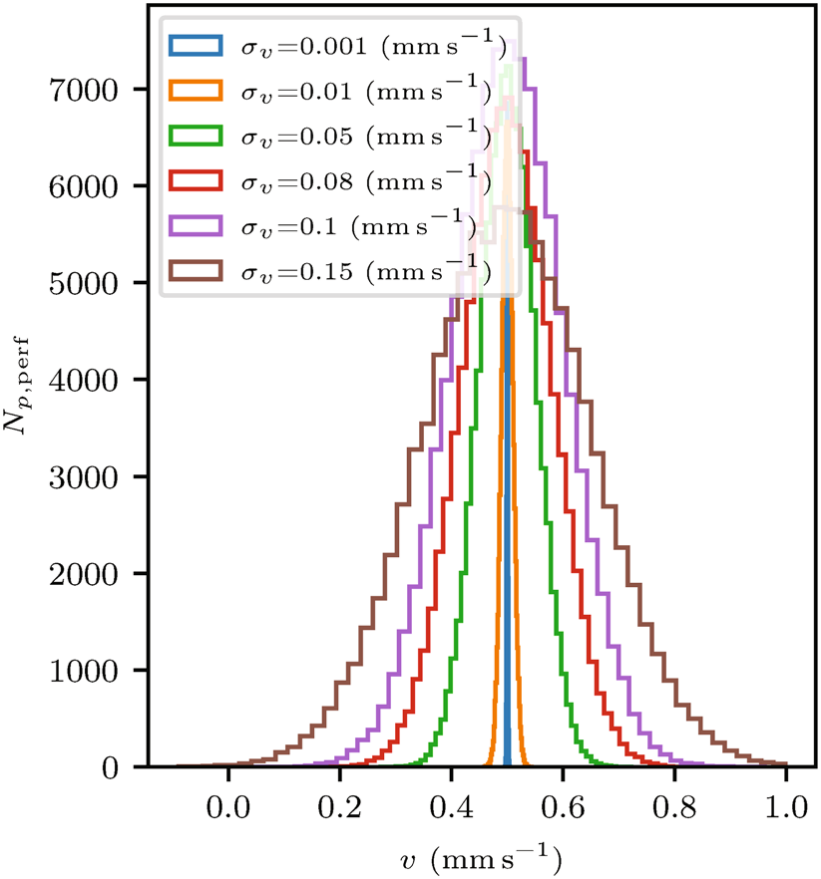
Histogram showing six Gaussian intercapillary velocity distributions for $N_{p,\text{perf}}=10^{5}$ perfusing spins. The Gaussian distributions are all centered around a constant mean inter‐capillary velocity $\bar{v}=0.5\;{\text{mm s}}^{-1}$.

Figure 3 illustrates the intercapillary Gaussian velocity distributions used in the simulations. Increased standard deviation $\sigma_{v}$ corresponds to greater velocity dispersion, while minimal $\sigma_{v}=0.001\;{\text{mm s}}^{-1}$ approximately models constant intercapillary velocity. A mean capillary velocity of $\bar{v}=0.5\;{\text{mm s}}^{-1}$ ^43^ has been assumed while the intercapillary velocity distributions have been truncated at −0.1 and $1\;{\text{mm s}}^{-1}$ to avoid extreme velocities.

The perfusing spins ($N_{p,\text{perf}}$) have been randomly seeded on one side of the voxel at plane z=0 and the perfusion simulations have been performed with $N_{p,\text{perf}}=10^{5}$ which we determined sufficient to reliably compute the perfusion signal (see Figure S4). In the perfusion model, particle displacements are exclusively influenced by the anisotropic model and rotation within the voxel, eliminating the need for a buffer zone.

The effect of perfusion on the diffusion tensor $D_{D}$ has been studied by computing a new tensor $D_{\mathrm{D+P}}$ fitting a mono‐exponential model to the total normalized signal calculated in Equation (7).

(8) $$\frac{S(b,\vec{e_{g}})}{S_{0}}=\exp(b{\vec{e_{g}}}^{T}D_{\mathrm{D+P}}\;\vec{e_{g}}).$$

Two different tensor fittings have been considered, with $b_{\text{ref}}$ values of 0 and $0.15\;\text{ms}\;\mu m^{-2}$, respectively. Additional simulations (see Table S3) suggested that using six encoding directions was enough to reliably calculate tensor parameters. Consequently, six diffusion encoding directions^44^ per *b*‐value were simulated resulting in a total of 12 combinations when using $b_{\text{ref}}=0.15\;\text{ms}\;\mu m^{-2}$ and seven directions using $b_{\text{ref}}=0\;\text{ms}\;\mu m^{-2}$. The mean and variance of the distribution of the eigenvectors has been calculated utilizing the procedure in Basser et al.^45^

### 2.8 Capillary network

Diffusion studies traditionally^21^ assumed an isotropic capillary segment distribution. However, the capillaries in skeletal muscle and in the myocardium are highly anisotropic with a preferred orientation aligned with the myocyte long axis.^24^ Several experimental studies have quantified the anisotropy in skeletal^29^, ^46^ and cardiac muscle^30^ capillaries and showed that a hemispherical Dimroth‐Watson axial distribution^47^, ^48^ provides a good fit to capillary segment orientation in both muscle types. On this basis, a Dimroth–Watson distribution has been used here for the zenith angle $\theta_{z}$ between each capillary segment and the cardiomyocyte long axis. The joint probability density of $p(\alpha ,\theta_{z}$) can be expressed as^48^

(9) $$\begin{array}{ll} p(\alpha ,\theta_{z}) & =\frac{1}{2\pi U_{0}}\sin\theta_{z}\exp\;(2K\cos^{2}\theta_{z})\\ U_{0} & =\int_{0}^{1}\exp\;(2Kx^{2})\;dx, \end{array}$$

and integrating with respect to the azimuth angle (α) reduces the equation to:

(10) $$p(\theta_{z})=\frac{1}{U_{0}}\sin\theta_{z}\exp\;(2K\cos^{2}\theta_{z}),$$

where K is a variable that determines the degree of anisotropy and may vary from isotropic K=0 to highly anisotropic K≫0. Note that due to the use of spherical coordinates the isotropic case K=0 distributes more points around the equator $\theta_{z}=\pi /2$ than closer to the cardiomyocytes axis $\theta_{z}=0$. Equation (10) assumes axial directions $\theta_{z}\in (0,\pi /2)$ and a uniform azimuth distribution $\alpha_{k}=U[0,2\pi ]$. A default value of K=3.25 has been chosen for the perfusion simulations as experimental data performed in rat hearts^30^ reports mean values of K=3.28 in the subepicardium and K=3.20 in the subendocardium. The length of the capillary segments has been obtained through a Weibull distribution with a mean and standard deviation of $\mu_{L}=60\;\mu m$ and $\sigma_{L}=40\;\mu m$.^24^, ^27^

## 3 RESULTS

### 3.1 The effects of ECV and variations in permeability

**FIGURE 4:**
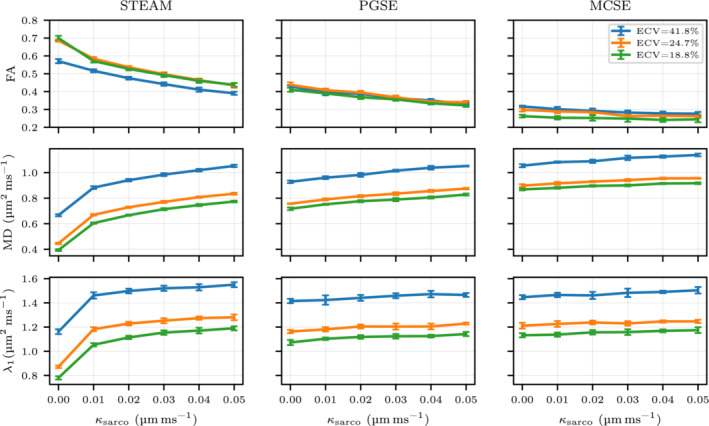
Fractional anisotropy, mean diffusivity, and $\lambda_{1}$ parameters of the diffusion tensor $D_{D}$ are illustrated considering three different extracellular volume fraction geometries (41.8%, 24.7%, 18.8%) and a range of realistic sarcolemma permeability values for cardiomyocytes. The graph shows mean values using six simulations with $N_{p}=10^{5}$ and $N_{t}=10^{4}$ walkers and timesteps respectively with a 95% confidence interval.

**FIGURE 5:**
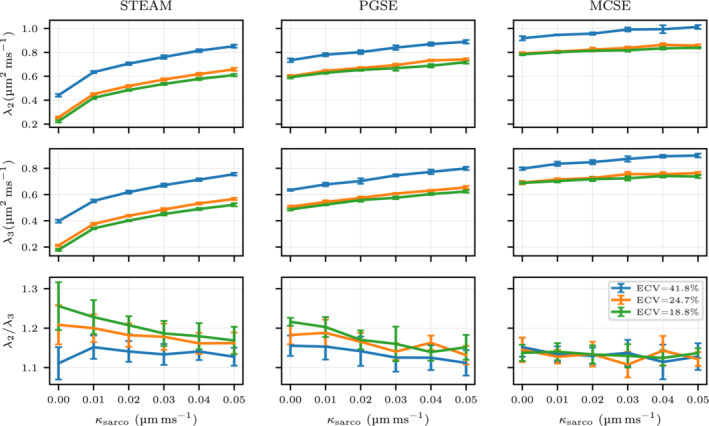
$\lambda_{2}$, $\lambda_{3}$ and $\lambda_{2}$/$\lambda_{3}$ are plotted for three different extracellular volume fraction geometries (41.8%, 24.7%, and 18.8%) and a range of realistic sarcolemma permeability values for cardiomyocytes. The plot illustrates mean values using six simulations with $N_{p}=10^{5}$ and $N_{t}=10^{4}$ walkers with the 95% confidence interval shown by the error bars.

The diffusion tensor is studied for changes in the ECV and sarcolemma permeability ($\kappa_{\text{sarco}}$). In Figures 4 and 5, the fractional anisotropy (FA), the mean diffusivity (MD) and each eigenvalue of the diffusion tensor are plotted for three different sequences (STEAM, PGSE, and MCSE). Increasing effects of restrictions on diffusion are observed for all three sequences when reducing the ECV from 41.8% to 18.8%. Conversely, increasing permeability reduces the anisotropy of the diffusion tensor, weakening FA and the $\lambda_{2}/\lambda_{3}$ ratio. Quantitatively, for the impermeable case $\kappa_{\text{sarco}}=0\;\mu {\text{m ms}}^{-1}$, this ECV reduction results in a decrease in MD of 40.9% (STEAM), 22.7% (PGSE) and 17.6% (MCSE). For ECV=24.7%, FA decreases from 0.7 to 0.5 as $\kappa_{\text{sarco}}$ rises from 0 to $0.03\;\mu {\text{m ms}}^{-1}$ for STEAM. The large MD reduction as ECV decreases observed for STEAM compared with the other sequences is mostly a consequence of large reductions in $\lambda_{2}$ and $\lambda_{3}$. Considering the same impermeable case, $\lambda_{2}$ only decreases 18.7% (PGSE) and 14.6% (MCSE) while $\lambda_{2}$ is reduced by 50% for STEAM. High $\lambda_{2}/\lambda_{3}$ ratios are observed for low ECV values when using STEAM and PGSE while this tendency is not present for MCSE as $\lambda_{2}/\lambda_{3}$ ratios are similar between the three ECV values.

Regarding the permeability, we observe a MD increase of 87.1% (STEAM), 15.62% (PGSE), and 6.5% (MCSE) and a $\lambda_{1}$ increase of 46.8% (STEAM), 5.6% (PGSE) and 2.9% (MCSE) for ECV=24.7% when increasing sarcolemma permeability from impermeable $\kappa_{\text{sarco}}=0\;\mu {\text{m ms}}^{-1}$ to the maximum value considered. There are small permeability‐related increases in the diffusivity in the direction of the primary diffusion eigenvector $\lambda_{1}$ for PGSE and MCSE. However, for STEAM, we observe a larger $\lambda_{1}$ increase of ≈34% for all ECV cases as $\kappa_{\text{sarco}}$ rises from 0 to $0.01\;\;\mu {\text{m ms}}^{-1}$. A more gradual increase in $\lambda_{2},\lambda_{3}$ when increasing permeability is noted for STEAM. Quantitatively, there is an increase of 156% for $\lambda_{2}$ (ECV=24.7%) and 168% for $\lambda_{3}$ (ECV=24.7%) when going from impermeable to the maximum permeability. These deviations are reduced to 29%, 25% and 8%, 7% for PGSE and MCSE, respectively. In the diffusion simulations, narrow uncertainty angle cones are obtained for all ECV and permeabilities values ranging from $0^{\circ }$ to $1^{\circ }$ for $\vec{E}1$ and $3^{\circ }$ to $6^{\circ }$ for $\vec{E}2,\vec{E}3$ suggesting that the eigenvectors are well‐defined for all three sequences (see Table S4).

### 3.2 ICD effect

**FIGURE 6:**
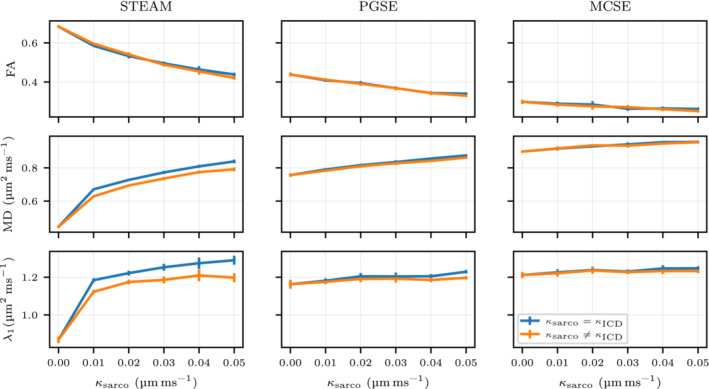
Illustration of the effect of intercalated disks (ICD) in three different sequences illustrating fractional anisotropy, mean diffusivity and $\lambda_{1}$. For this plot, an extracellular volume fraction=24.7% and a $\kappa_{\text{sarco}}=0.05\;\mu {\text{m ms}}^{-1}$ have been considered. The different plots show mean values using six diffusion simulations with $N_{p,\text{diff}}=10^{5}$ and $N_{t}=10^{4}$ walkers with a 95% confidence interval. Note that we have considered $\kappa_{\text{ICD}}=0.005\;\mu {\text{m ms}}^{-1}$ for all $\kappa_{\text{sarco}}$ cases except for the impermeable case where $\kappa_{\text{ICD}}=\kappa_{\text{sarco}}=0\;\mu {\text{m ms}}^{-1}$ has been considered.

The effect of ICD permeability is investigated by introducing a lower permeability on the endcaps of the simulated cardiomyocytes. Figure 6 illustrates the effect of reduced ICD permeability ($\kappa_{\text{ICD}}$) compared to the results obtained when considering a homogenous permeability across all membranes of the cardiomyocytes. The lower ICD permeability predominantly reduces the primary eigenvalue $\lambda_{1}$ (7% reduction for $\kappa_{\text{sarco}}=0.05\;\;\mu {\text{m ms}}^{-1}$) which in turn reduces the MD for STEAM. This effect is only apparent when $\kappa_{\text{sarco}}\neq 0\;\mu {\text{m ms}}^{-1}$ and only for the STEAM sequence. No major changes in FA, $\lambda_{2}$ and $\lambda_{3}$ related to ICD permeability are observed for any of the three sequences (see Figure S5).

### 3.3 Perfusion simulations

**FIGURE 7:**
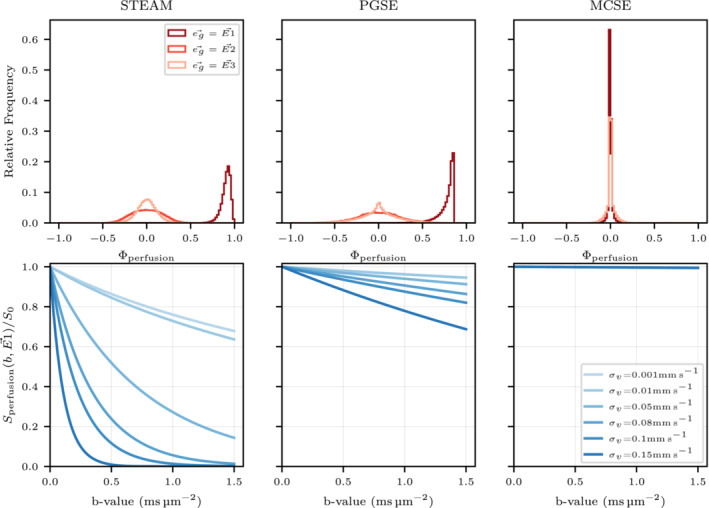
Top figures: The relative particle frequency for the normalized phase $\Phi_{\text{perfusion}}$ is plotted for three different gradient directions parallel to the eigenvectors of the diffusion tensor D. Bottom figures: The signal attenuation along $\vec{E1}$ is plotted for the three sequences considering several Gaussian intercapillary velocities varying its SD from $\sigma_{v}=0.001{\text{mm s}}^{-1}$ to $\sigma_{v}=0.15\;{\text{mm s}}^{-1}$.

Figure 7 depicts the normalized phase $\Phi_{\text{perfusion}}$ described in Equation (6) when the diffusion encoding gradient direction is aligned with each of the three diffusion tensor eigenvectors ($\vec{E}1,\vec{E}2,\vec{E}3$). A narrow phase distribution is noted for STEAM and PGSE when the gradient direction is parallel to the main capillary direction ($\vec{E}1$, which is also the mean cardiomyocyte orientation) while encoding along the two orthogonal directions ($\vec{E}2,\vec{E}3$) results in wider symmetric distributions. Encoding along the main capillary direction with minimal inter‐capillary velocity dispersion $\sigma_{v}=0.001\;{\text{mm s}}^{-1}$ results in the maximum total phase (i.e., all magnetization vectors point in a similar direction) and the highest normalized perfusion signal $S_{\text{perfusion}}(b,\vec{e_{g}})/S_{0}$, while encoding along the orthogonal directions leads to signal loss reducing the normalized perfusion signal. For MCSE, the normalized phase distributions show a peak centered at 0 for all three gradient directions indicating relative insensitivity to mean capillary flow velocity changes in the intercapillary velocity dispersion while the narrow width of the same $\Phi_{\text{perfusion}}$ distributions result in minimal perfusion signal loss in all directions. For STEAM and PGSE, increasing the intercapillary velocity dispersion $\sigma_{v}>>0$ broadens the phase $\phi_{\text{perfusion}}$ distribution resulting in reduced normalized perfusion signals. As an example, using STEAM the normalized perfusion signal ($S_{\text{perfusion}}(b,\vec{E}1)/S_{0}$) in the direction of $\vec{E}1$ considering a *b*‐value of $0.15\text{ms}\mu m^{-2}$ is reduced from 0.951 to 0.234 when the velocity dispersion is increased from $\sigma_{v}=0.001\;{\text{mm s}}^{-1}$ to $\sigma_{v}=0.15\;{\text{mm s}}^{-1}$.

**FIGURE 8:**
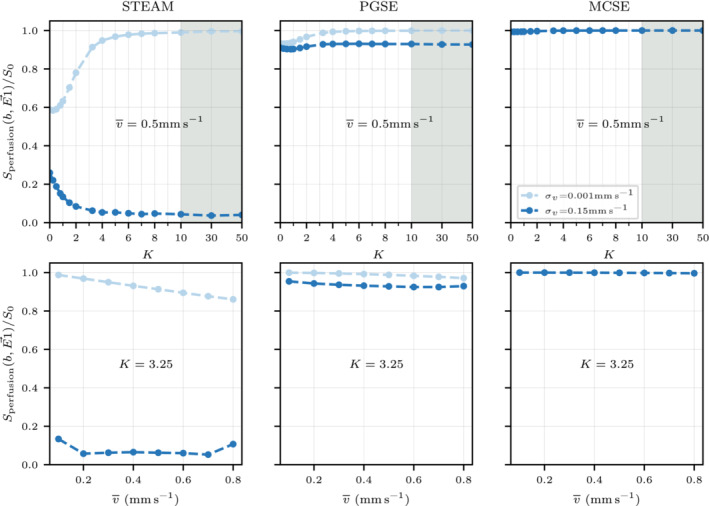
The signal attenuation along the $\vec{E}1$ direction for a *b*‐value of $b=0.6\;\text{ms}\;\mu m^{-2}$ is plotted for the two extreme intercapillary velocity dispersions $\sigma_{v}=0.001$ and $\sigma_{v}=0.15$ varying the capillary network anisotropy K (top row) and the mean capillary velocity $\bar{v}$ (bottom row). A constant $\bar{v}=0.5.\;{\text{mm s}}^{-1}$ and a constant K=3.25 has been considered when altering K and $\bar{v}$, respectively. Note that the gray region in the three top figures indicates two different scales in the x‐axis showcasing extreme anisotropic cases.

Figure 8 illustrates the effect of the capillary anisotropy K and the mean intercapillary velocity $\bar{v}$ on the normalized perfusion signal measured in the direction parallel to the cardiomyocyte long axis. Even with a spherical distribution, the isotropic regime K=0 results in different perfusion signal values for both velocity distributions $\sigma_{v}$. Increasing the anisotropy of the capillary network K>0 increases the normalized perfusion signal and results in almost no signal loss when the particles travel at a constant velocity $\sigma_{v}=0.001\;{\text{mm s}}^{-1}$ while using a higher intercapillary velocity dispersion reduces the normalized perfusion signal. Note that these signal differences are greater for long diffusion time sequences. Quantitatively, the normalized perfusion signal at *K* = 3.25 is reduced from 0.91 to 0.053 and from 0.98 to 0.93, respectively, for STEAM and PGSE when increasing the inter‐capillary velocity dispersion $\sigma_{v}$ from 0.001 to 0.15. STEAM and PGSE are less sensitive to changes in $\bar{v}$ than in **s**_*v*_ and K and small deviations in perfusion signal are only observed when the intercapillary velocity distribution is near constant ($\sigma_{v}=0.001\;{\text{mm s}}^{-1}$). Similarly to the results in Figure 7, the normalized signal from MCSE displays minimal changes in response to the changes in mean inter‐capillary velocity $\bar{v}$, the velocity dispersion $\sigma_{v}$ or the anisotropy K of the capillary network.

### 3.4 Perfusion and diffusion

**FIGURE 9:**
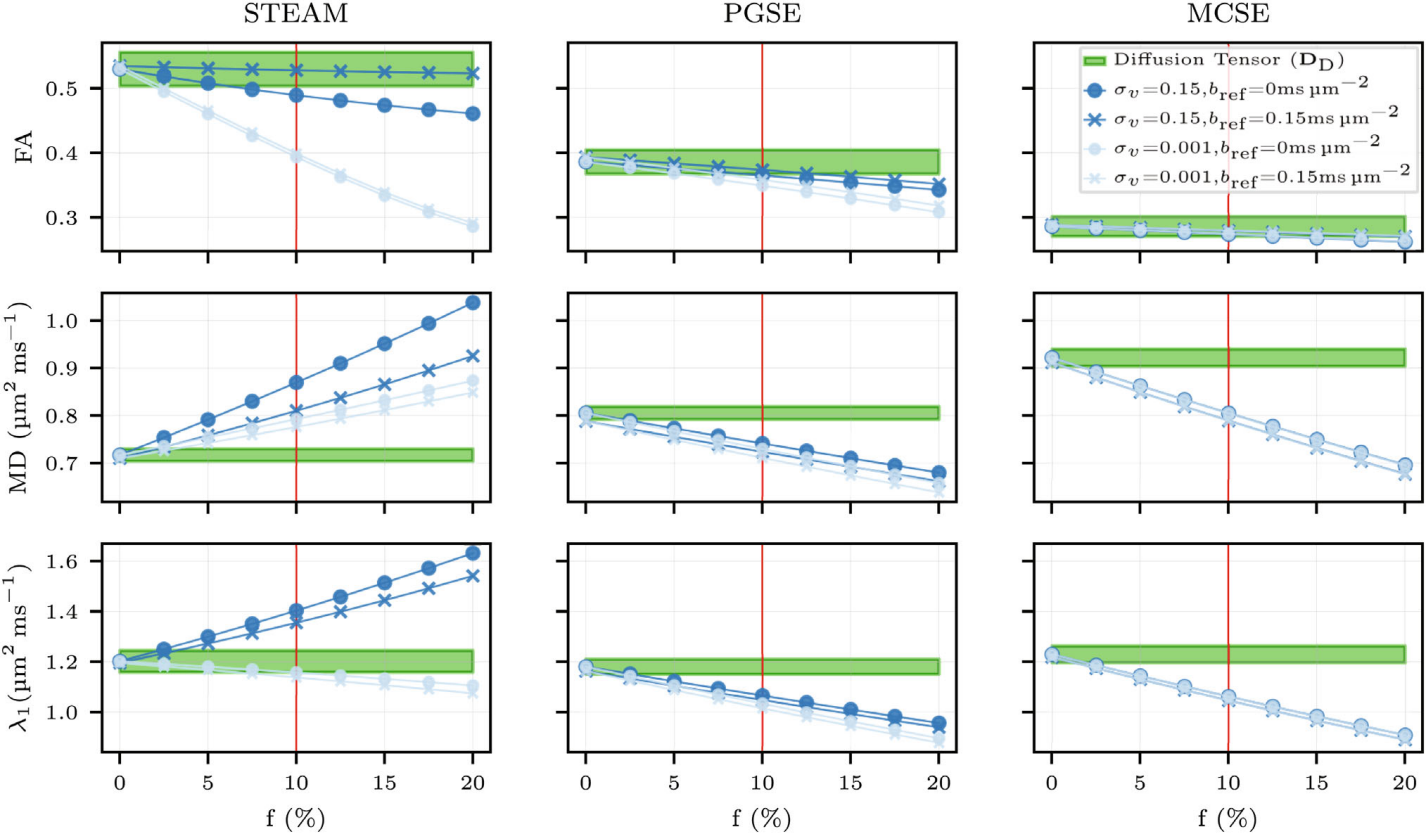
Fractional anisotropy, mean diffusivity, and $\lambda_{1}$ parameters for several perfusion fraction values using a K=3.25 (red vertical line in Figure 10) representative of the capillary network in the myocardium. The green area shows the 95% confidence interval obtained in the diffusion simulations without the effect of perfusion. Two extreme inter‐capillary velocities are plotted considering two distinct fittings tensor methods with $b_{\text{ref}}=0\;\text{ms}\;\mu m^{-2}$ and $b_{\text{ref}}=0.15\;\text{ms}\;\mu m^{-2}$. The diffusion results have been obtained considering six simulations of $10^{5}$ particles and $10^{4}$ time steps while the perfusion simulations have been performed using only one simulation of $10^{5}$ particles.

Figure 9 illustrates $D_{\mathrm{D+P}}$ tensor parameters for several f values considering a constant K=3.25 and $\bar{v}=0.5\;{\text{mm s}}^{-1}$. The tensor fitting is performed with two different reference *b*‐values ($b_{\text{ref}}=0\;\text{ms}\mu m^{-2}$, $b_{\text{ref}}=0.15\;\text{ms}\mu m^{-2}$) considering two extreme inter‐capillary velocity distributions ($\sigma_{v}=0.001\;{\text{mm s}}^{-1}$, $\sigma_{v}=0.15\;{\text{mm s}}^{-1}$). The MD, $\lambda_{1,2,3}$, and FA are linearly reduced as the perfusion fraction increases for PGSE and MCSE while STEAM presents higher apparent diffusivity in all diffusion directions (see Figure S6). The eigenvalue reductions observed in both short diffusion encoding sequences are the same for both tensor calculations and both extreme inter‐capillary velocity distributions $\sigma_{v}$. Quantitatively, for both $\sigma_{v}$ and tensor calculations, MD decreases ≈23% for PGSE and ≈35.6% for MCSE when f rises from 0% to 20%. Despite these large changes in MD, the FA maintains a value close to the diffusion only result (f=0%).

Due to a longer diffusion time, the effect of perfusion when increasing f(%) is greater on STEAM than PGSE or MCSE amplifying all the eigenvalues of the tensor $\lambda_{1,2,3}$. For STEAM, a distinction between the two extreme intercapillary velocity distributions $\sigma_{v}$ is observed in the tensor parameters assessed. A small velocity dispersion $\sigma_{v}=0.001\;{\text{mm s}}^{-1}$ only increases $\lambda_{2,3}$, maintaining the same diffusivity along the cardiomyocytes long axis direction ($\lambda_{1}$) and reducing the overall tensor FA. On the contrary, as observed in Figure 7, increasing $\sigma_{v}$ for STEAM yields maximum signal loss toward $\vec{E}1$ intensifying the overall measured eigenvalues in all three principal directions resulting in higher MD and lower FA values. Conversely to PGSE and MCSE, larger variations are observed between the tensor fittings with the two different reference *b*‐values $b_{\text{ref}}$ for STEAM. The tensor fitting with $b_{\text{ref}}=0.15\;\text{ms}\mu m^{-2}$ results in FA, MD, and $\lambda_{1}$ values closer to the equivalent results from the diffusion only simulation. When $\sigma_{v}=0.15\;{\text{mm s}}^{-1}$ the use of $b_{\text{ref}}=0.15\;\text{ms}\mu m^{-2}$ in STEAM removes the effects of perfusion from the FA. For STEAM, f=10% and $\sigma_{v}=0.15\;{\text{mm s}}^{-1}$ the tensor fitting using $b_{\text{ref}}=0.15\;{\text{mm s}}^{-1}$ reduces the MD by 8.9% and increases the FA by 10.76% compared to $b_{\text{ref}}=0\;{\text{mm s}}^{-1}$.

**FIGURE 10:**
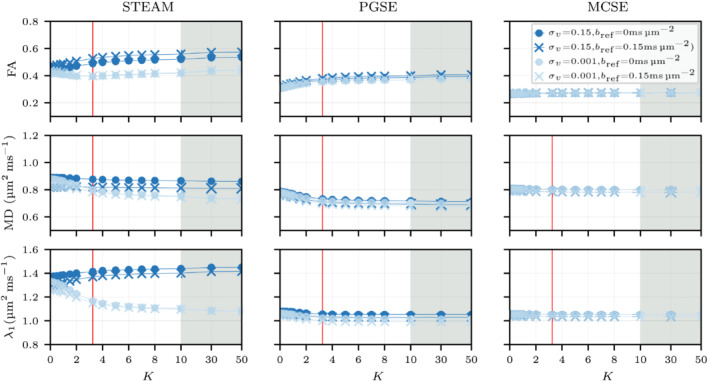
Fractional anisotropy, mean diffusivity, and $\lambda_{1}$ parameters considering a constant perfusion fraction of f=10% (red vertical line in Figure 9) for a range of different capillary networks ranging from an isotropic (K=0) to highly anisotropic (K>10) capillary distributions toward the long cardiomyocytes axis. Two extreme intercapillary velocities are depicted considering two distinct fitting tensors methods with $b_{\text{ref}}=0\;\text{ms}\mu m^{-2}$ and $b_{\text{ref}}=0.15\;\text{ms}\mu m^{-2}$. Note that the red line depicts a realistic anisotropic value K=3.25 for the capillary network observed in the myocardium and the gray area illustrates extreme anisotropic cases in a different horizontal axis scale.

Figure 10 investigates the effect of varying the anisotropy of the capillary network. Similarly to Figure 9, two extreme intercapillary velocity distributions $\sigma_{v}$ are considered together with tensor fittings with two different reference *b*‐values. Note that the differences observed for STEAM in the normalized perfusion signal when the capillary network is isotropic K=0 in Figure 8 (top row) are highly reduced in $\lambda_{1}$ (see Figure 10) when adding the normalized diffusion signal. Tensor variations are mostly observed for STEAM and PGSE when 0<K<10, while differences in the tensor due to changes in $\sigma_{v}$ are only observed for STEAM. Specifically for this sequence, increasing $\sigma_{v}$ reduces the influence of K on $\lambda_{1}$. Quantitatively, $\lambda_{1}$ decreases 21% for $\sigma_{v}=0.001\;{\text{mm s}}^{-1}$ and only 3% for $\sigma_{v}=0.15\;{\text{mm s}}^{-1}$ as K increases from 0 (isotropic network) to 50 (highly anisotropic network).

## 4 DISCUSSION

Simulations of the effects of cell membrane permeability and capillary perfusion on DT‐CMR show a significant dependence on model parameters. Three sequences (STEAM, PGSE, and MCSE) were simulated using MC random walk^20^ on a histology‐based tissue microstructure^10^ in which the manually segmented cardiomyocytes were morphed (shrunk and thickened) to represent a physiological range of ECVs (18.8% to 41.8%). While STEAM and MCSE are in vivo and ex vivo DT‐CMR techniques, PGSE is only suitable for ex vivo but is included for completeness and scientific interest.

The primary eigenvector of the diffusion tensor ($\vec{E}1$) is aligned with the cardiomyocytes long axis^10^ while the second and third eigenvector directions ($\vec{E}2,\vec{E}3$) are contained in perpendicular plane to $\vec{E}1$, with $\vec{E}2$ lying in the sheetlet plane and $\vec{E3}$ defining the sheetlet structure as its normal.^31^, ^49^ Having $\lambda_{2}>>\lambda_{3}$ and narrow cones of uncertainty suggests that there is a preferred diffusion direction in the perpendicular plane to $\vec{E}1$ due to a specific sheetlet geometry structure. The results indicate that STEAM and PGSE have more sensitivity to the sheetlet structure compared to MCSE as low ECV values are linked to higher $\lambda_{2}/\lambda_{3}$ ratios. For all permeability values, all sequences show a reduction in MD with decreasing ECV due to a lower diffusion coefficient in the intracellular space, $D_{\text{ICS}}<D_{\text{ECS}}$. PGSE and STEAM show higher FA values for low ECV fractions while MCSE demonstrates the opposite. Due to the short effective diffusion time of the MCSE sequence the ratio $\lambda_{2}/\lambda_{3}$ is the same for all the three extracellular volume geometries and the anisotropy of the tensor (FA) is mostly driven by $\lambda_{1}$.

The diffusion restriction can be reduced by including permeability in the diffusion simulations allowing the walkers to cross between compartments. The sensitivity of each sequence to permeability is related to the comparison between the diffusion time (Δ) and the exchange time. The exchange time ($\tau_{\text{exchange}}$) is directly proportional to ECV and inversely proportional to the permeability.^50^ Small permeability‐related deviations in DT‐CMR parameters are observed for PGSE and MCSE. However, major deviations are observed for STEAM, particularly at low permeability values. A permeability of $\kappa_{\text{sarco}}=0.01\;\mu m\;{\text{ms}}^{-2}$ is linked to a water exchange time of 96 ms for the highest ECV. Note that for STEAM (Δ≈𝒪(1000ms)) there is ample time for the walkers to cross multiple membranes between ***ICS and ECS***, in contrast to the short diffusion time of both PGSE and MCSE. This explains the sudden jump in $\lambda_{1}$, MD, and FA when increasing permeability $\kappa_{\text{sarco}}$ from 0μm to $0.01\;\mu {\text{m ms}}^{-1}$, whereas a more gradual transition is observed for PGSE and MCSE as $\Delta <\tau_{\text{exchange}}$.

ICDs connect neighboring cardiomyocytes and enable the passage of ions between cells allowing cardiac action potential to spread along the cardiac muscle.^51^ Abnormalities of impulse propagation, arrhythmic tendency and hypertrophied myocardium have been linked to significant changes in gap junctions.^52^ To study the sensitivity of the diffusion tensor to the presence of the ICD, results are compared for constant sarcolemma permeability with results for lowered ICD permeability^12^ ($\kappa_{\text{ICD}}$). Among all the sequences evaluated only STEAM shows changes in the diffusion tensor in response to reduced ICD permeability, as the spins diffuse further for larger Δ and are more likely to collide with the cardiomyocyte end caps. Short diffusion time sequences limit the number of collisons with the ICD zones reducing any observable effect.

The perfusion simulations have been performed by recreating an anisotropic capillary network with the mean capillary orientation aligned with the cardiomyocytes long axis ($\vec{E}1$). The perfusion simulations are performed separately from the diffusion simulations but considering the same HA rotation within the voxel. The simplicity of the perfusion model permits computing a phase for each particle that is normalized ($\Phi_{\text{perfusion}}$) by the *b*‐value, the constant capillary velocity and the overall sequence time. The signal at the end of the simulation is obtained by summing the contributions of each particle's magnetization vector which are modified by the phase distribution $\phi_{\text{perfusion}}$.

In order to simulate a distribution of velocities within the capillary network, a Gaussian intercapillary velocity distribution has been assumed with SDs between $\sigma_{v}=0.001\;{\text{mm s}}^{-1}$ and $\sigma_{v}=0.15\;{\text{mm s}}^{-1}$. Note that if $\sigma_{v}=0.001\;{\text{mm s}}^{-1}$ the Gaussian bell is narrow, approximating a constant velocity which leads to similarly narrow distribution for ($\phi_{\text{perfusion}}$) and the normalized phase $\Phi_{\text{perfusion}}$. When the diffusion encoding gradient is parallel to $\vec{E1}$ and $\sigma_{v}=0.001\;{\text{mm s}}^{-1}$, due to the similarity between $\phi_{\text{perfusion}}$ and $\Phi_{\text{perfusion}}$, there is minimal perfusion signal loss as the phase dispersion within a voxel is low, that is, all the magnetization vectors point in a similar direction. This is in line with the results reported in a similar perfusion model.^27^ However, the findings^27^ of minimal signal loss due to perfusion in the direction of $\vec{E}1$ contradict experimental studies^26^ and other numerical simulations^28^ which suggest that the direction $\vec{E}1$ is related to the maximum signal loss.

The present results demonstrate the importance of considering variable velocities within the capillary network as this spreads and changes the normalized phase $\Phi_{\text{perfusion}}$ distribution. The perfusion simulations show that incrementing the intercapillary velocity dispersion from $\sigma_{v}=0.001\;{\text{mm s}}^{-1}$ to $\sigma_{v}=0.15\;{\text{mm s}}^{-1}$ yields a faster perfusion signal reduction with *b*‐value in the $\vec{E}1$ direction and shifts the maximum signal decrease toward this direction. These results are in line with a numerical study that reported faster signal reduction for laminar flows compared to plug flows for the same capillary networks.^26^ As shown in Figure 7, large intercapillary velocity dispersions $\sigma_{v}=0.15\;{\text{mm s}}^{-1}$ reduce the effect of the anisotropy of capillary network or the mean magnitude of the intercapillary velocity to changes in perfusion signal. Conversely, a capillary network based on the original intravoxel incoherent motion method^21^ (isotropic) is less susceptible to changes in the intercapillary velocity dispersion. Nevertheless, small variations in anisotropy (K≈2) in the capillary network when using long diffusion time sequences present large deviations from the isotropic case. We argue therefore that it is necessary to consider both dispersion in orientation and in velocity. However, the present perfusion model is limited to constant intracapillary velocities and thus MCSE shows minimal sensitivity to perfusion.

The effect of perfusion on DT‐CMR has been simulated by fitting a tensor $D_{\text{D+P}}$ to a weighted signal from both the diffusion and perfusion processes. The impact of perfusion on the tensor $D_{\text{D+P}}$ depends on the perfusion fraction f(%) and the capillary network geometry. The tensor $D_{\text{D+P}}$ has been computed considering a *b*‐value of $0.6\;\text{ms}\mu m^{-2}$ and two different $b_{\text{ref}}$ values of $0\;\text{ms}\mu m^{-2}$ and $0.15\;\text{ms}\mu m^{-2}$. Long diffusion time sequences (STEAM) allow water molecules to perfuse and disperse large distances, increasing the sensitivity of the perfusion signal to changes in $\sigma_{v}$ while less sensitivity is observed for short diffusion time sequences (PGSE and MCSE). As demonstrated in Figure 7, for STEAM when the gradient is parallel to $\vec{E}1$, increasing $\sigma_{v}$ results in a rapid decline in the normalized perfusion signal as *b*‐value increases. When the *b*‐value is fixed and $\sigma_{v}$ is large, the signal loss from perfusion in STEAM is greater than that of diffusion, lowering the total signal resulting in increased eigenvalues and MD. Conversely, the total signal for PGSE and MCSE increases because the signal loss associated with diffusion is greater than that of perfusion. This results in a counter‐intuitive reduction in MD as the perfusion fraction (*f*) increases as the proportion of signal originating from perfusing spins increases in comparison to diffusing spins.

For STEAM, an increased reference *b*‐value $b_{\text{ref}}=0.15\;\text{ms}\mu m^{-2}$ reduces the effect of perfusion leading toward similar FA values for both $D_{\text{D+P}}$ and $D_{D}$ tensors. Similarly an increase in the reference *b*‐value in experimental studies^53^ is thought to reduce the effect of perfusion. When using STEAM, increasing the inter‐capillary velocity dispersion ($\sigma_{v}$) not only accelerates the perfusion signal decay with *b*‐value as described above, but reduces the contribution of the anisotropy of the capillary network. This can be observed in Figure 10 for changes in $\lambda_{1}$ where STEAM and $\sigma_{v}=0.15\;{\text{mm s}}^{-1}$ show moderate changes in the tensor (of the order of 3% for $\lambda_{1}$) between K=0 and K≫0 while larger deviations are observed for $\sigma_{v}=0.001\;{\text{mm s}}^{-1}$.

## 5 CONCLUSION

The separate and combined effects of permeability and microvascular perfusion on DT‐CMR measurements have been investigated with the extent of possible confounding contributions due to altered diffusion at the ICD. The models developed in this work provide new insights into the response of DT‐CMR to microstructural changes underlying cardiac pathology, which will be of great relevance in interpreting changes observed in clinical DT‐CMR studies.

In conclusion, ECV and permeability have measurable impacts on DT‐CMR parameters. Increasing permeability reduces the exchange time between the water molecules located in the intracellular and ECS, augmenting the effective diffusion. The ratio of the water exchange and diffusion times determines the sensitivity of a certain sequence to changes in permeability. Of the sequences considered, results show that STEAM is the most sensitive to permeability variations and the only sequence with measurable variations in $\lambda_{1}$ in response to changes in the permeability of the ICD. These findings suggest that STEAM is a potential sequence for the early detection of certain permeability‐related pathologies and abnormalities of impulse propagation.

With regard to microvascular perfusion, it is concluded that the intercapillary velocity dispersion ($\sigma_{v}$) has a major impact on the perfusion signal when capillary networks are anisotropic. Long diffusion time sequences (STEAM) are more sensitive to changes in $\sigma_{v}$ as water molecules perfuse further during the diffusion time and over more tortuous paths. Our results show that STEAM yields the highest perfusion signal loss toward the primary diffusion eigenvector $\vec{E}1$ for the maximum intercapillary velocity dispersion considered. In contrast, PGSE and MCSE are less sensitive to perfusion leading to a more pronounced diffusion signal loss compared to perfusion. This results in an unexpected decrease in MD and all eigenvalues in $D_{\text{D+P}}$ as the perfusion fraction is increased for PGSE and MCSE sequences.

For all sequences but specifically for STEAM, it is shown that that using a tensor fitting with a $b_{\text{ref}}>0\;\text{ms}\mu m^{-2}$ reduces the effect of perfusion when comparing the diffusion tensor $D_{D}$ parameters to $D_{\text{D+P}}$. For clinical DT‐CMR studies where changes in permeability may be of interest, STEAM should be considered the sequence of choice. However, the sensitivity of STEAM to microvascular perfusion must also be considered and these confounding effects can be at least partially mitigated by the use of a higher reference *b*‐value.

## CONFLICT OF INTEREST

Dudley J. Pennell: Research funding from Siemens. Speakers honoraria from Bayer and Circle.

## Supporting information




**Figure S1.** Comparison between Finite Element solution and Random Walk in a representative domain for $\kappa_{sarco}=0.05\;\mu m\;{\text{ms}}^{-2}$ and T=500 ms. The FE solution was obtained considering Continuous Galerkin (CG) elements while assuming a small buffer region accounting for the membrane.
**Figure S2.** Comparison between Finite Element solution and Random Walk in a representative domain for $\kappa_{sarco}=0\;\mu m\;{\text{ms}}^{-2}$ and T=500 ms. The FE solution was obtained considering Continuous Galerkin (CG) elements while assuming a small buffer region accounting for the membrane.
**Figure S3.** Illustration of three different extracellular volume fractions (ECV) obtained by morphing the manually segmented cardiomyocytes.
**Figure S4.** Convergence of the perfusion signal obtained for a *b*‐value of $0.6\;{\text{ms ms}}^{-2}$ for different number of perfusion particles $N_{p,\text{perf}}$.
**Figure S5.** Illustration of the effect of ICD in three different sequences illustrating $\lambda_{2}$ and $\lambda_{3}$. For this plot, an ECV=24.7% and a $\kappa_{\text{sarco}}=0.05\;\mu {\text{m ms}}^{-1}$ have been considered. The different plots show mean values using 6 diffusion simulations with $N_{p,\text{diff}}=10^{5}$ and $N_{t}=10^{4}$ walkers with a 95% confidence interval. Note that we have considered $\kappa_{\text{ICD}}=0.005\;\mu m\;{\text{ms}}^{-2}$ for all $\kappa_{\text{sarco}}$ cases except for the impermeable case where $\kappa_{\text{ICD}}=\kappa_{\text{sarco}}=0\;\mu m\;{\text{ms}}^{-2}$ has been considered.
**Figure S6.** $\lambda_{2}$ and $\lambda_{3}$ parameters for several perfusion fraction values using a K=3.25 (red vertical line representative of the capillary network in the myocardium). The green area shows the 95% confidence interval obtained in the diffusion simulations without the effect of perfusion. Two extreme inter‐capillary velocities are plotted considering two distinct fittings tensor methods with $b_{\text{ref}}$ = $0\;\text{ms}\mu m^{-2}$ and $b_{\text{ref}}$ = $0.15\;\text{ms}\mu m^{-2}$. The diffusion results have been obtained considering 6 simulations of $10^{5}$ particles and $10^{4}$ time steps while the perfusion simulations have been performed using only 1 simulation of $10^{5}$ particles.
**Figure S7.** The perfusion and diffusion signal attenuations along $\vec{E1}$ are plotted for the three sequences considering several Gaussian inter‐capillary velocities varying its SD from $\sigma_{v}=0.001\;{\text{mm s}}^{-1}$ to $\sigma_{v}=0.15\;{\text{mm s}}^{-1}$.
**Table S1.** Comparison of diffusion tensor parameters acquired from 6 diffusion simulations using an anisotropic and isotropic voxel. The diffusion simulations are performed considering STEAM, ECV = 24.7%, and $\kappa_{\text{sarco}}=0.02\;\mu m\;{\text{ms}}^{-2}$. The results show similar results using the anisotropic and isotropic voxels which can be attributed to the similarity in microstructure in the slice direction.
**Table S2.** Sequence parameters for the three typical DT‐CMR sequences considered in the simulations. δ denotes the gradient flat‐top durations and ϵ the ramp‐up and ramp‐down time. $G_{\max,b_{\text{ref}}}$ refers to a reference *b*‐value of $0.15\;\text{ms}\mu m^{-2}$ and $G_{\max,b}$ to a *b*‐value of $0.6\;\text{ms}\mu m^{-2}$.
**Table S3.** Comparison of diffusion tensor parameters acquired from 6 diffusion simulations using various sets of directions, illustrating consistent results across different direction configurations. Simulations are performed considering STEAM, ECV = 24.7%, and $\kappa_{\text{sarco}}=0.02\;\mu m\;{\text{ms}}^{-2}$.
**Table S4.** Comparison of cones of uncertainty for MCSE, PGSE and STEAM sequences for the second and third eigenvectors across two distinct extreme permeabilities.
