# Supplementary material for: Realistic numerical simulations of diffusion tensor cardiovascular magnetic resonance: The effects of perfusion and membrane permeability
Source: Magn Reson Med. 2023 Jul 6;90(4):1641–56. doi: 10.1002/mrm.29737 (PMC10952789; doi:10.1002/mrm.29737)
Supplement: Supplementary file 1 — Figure S1. Comparison between Finite Element solution and Random Walk in a representative domain for κsarco=0.05μmms−2 and T=500 ms. The FE solution was obtained considering Continuous Galerkin (CG) elements while assuming a small buffer region accounting for the membrane. Figure S2. Comparison between Finite Element solution and Random Walk in a representative domain for κsarco=0μmms−2 and T=500 ms. The FE solution was obtained considering Continuous Galerkin (CG) elements while assuming a small buffer region accounting for the membrane. Figure S3. Illustration of three different extracellular volume fractions (ECV) obtained by morphing the manually segmented cardiomyocytes. Figure S4. Convergence of the perfusion signal obtained for a b‐value of 0.6ms ms−2 for different number of perfusion particles Np,perf. Figure S5. Illustration of the effect of ICD in three different sequences illustrating λ2 and λ3. For this plot, an ECV=24.7% and a κsarco=0.05μm ms−1 have been considered. The different plots show mean values using 6 diffusion simulations with Np,diff=105 and Nt=104 walkers with a 95% confidence interval. Note that we have considered κICD=0.005μmms−2 for all κsarco cases except for the impermeable case where κICD=κsarco=0μmms−2 has been considered. Figure S6. λ2 and λ3 parameters for several perfusion fraction values using a K=3.25 (red vertical line representative of the capillary network in the myocardium). The green area shows the 95% confidence interval obtained in the diffusion simulations without the effect of perfusion. Two extreme inter‐capillary velocities are plotted considering two distinct fittings tensor methods with bref = 0msμm−2 and bref = 0.15msμm−2. The diffusion results have been obtained considering 6 simulations of 105 particles and 104 time steps while the perfusion simulations have been performed using only 1 simulation of 105 particles. Figure S7. The perfusion and diffusion signal attenuations along E1→ are plotted for the three seque [file MRM-90-1641-s001.pdf]

Voxel type	FA	MD	λ_1	λ_2	λ_3
Anisotropic Voxel (current model, 2800×2800× 8000μm^3)	0.5250 ±0.0141	0.7265 ±0.0074	1.2120 ±0.0231	0.5231 ±0.0092	0.4445 ±0.0102
Isotropic Voxel (2800 × 2800 × 2800μm^3)	0.5346 ±0.0115	0.7371 ±0.0071	1.2405 ±0.0139	0.5272 ±0.0119	0.4436 ±0.0089

TABLE S1 Comparison of diffusion tensor parameters acquired from 6 diffusion simulations using an anisotropic and isotropic. 6 diffusion simulations were performed considering STEAM, ECV=24.7%, and $\kappa_{\text{sarco}} = 0.02\,\mu\text{m ms}^{-1}$. The results show similar results using the anisotropic and isotropic voxels which can be attributed to the similarity in microstructure in the slice direction.

Parameters	PGSE	MCSE	STEAM
$G_{\text{max},b_{\text{ref}}}$ (mT m^{-1})	26.18	23.92	22.18
$G_{\text{max},b}$ (mT m^{-1})	53.63	47.85	44.36
Δ (ms)	19.3	23.92	1000
ϵ (ms)	0.89	0.89	0.89
δ (ms)	8.62	7.22 and 15.33	0.57

TABLE S2 Sequence parameters for the three typical DT-CMR sequences considered in the simulations. δ denotes the gradient flat-top durations and ϵ the ramp-up and ramp-down time. $G_{\text{max},b_{\text{ref}}}$ refers to a reference b-value of 0.15

.

Number of Directions	FA	MD	λ_1	λ_2	λ_3
6 directions (cuboidal diagonal directions, used in the simulations)	0.5250 ±0.014	0.7265 ±0.0074	1.2120 ±0.0231	0.5231 ±0.0092	0.4445 ±0.0102
6 directions (Electrostatic repulsion distribution)	0.5260 ±0.014	0.7262 ±0.0074	1.2125 ±0.0226	0.5230 ±0.009	0.4431 ±0.0110
30 directions (Electrostatic repulsion distribution)	0.5263 ±0.0142	0.7262 ±0.074	1.2129 ±0.0229	0.5229 ±0.0094	0.4427 ±0.0109
60 directions (Electrostatic repulsion distribution)	0.5263 ±0.0142	0.7262 ±0.074	1.2129 ±0.0229	0.5229 ±0.0094	0.4427 ±0.0109

TABLE S3 Comparison of diffusion tensor parameters acquired from 6 diffusion simulations using various sets of directions, illustrating consistent results across different direction configurations. Simulations were performed considering STEAM, ECV=24.7%, and $\kappa_{\text{sarco}} = 0.02\,\mu\text{m ms}^{-1}$.

Sequence	θ_{cone} ($\vec{E2}_{\text{sim}}$, $\vec{E2}_{\text{mean}}$)	θ_{cone} ($\vec{E3}_{\text{sim}}$, $\vec{E3}_{\text{mean}}$)
MCSE ($\kappa_{\text{sarco}} = 0\,\mu\text{m ms}^{-1}$)	1.96 ±1.3	1.46 ±0.8
MCSE ($\kappa_{\text{sarco}} = 0.05\,\mu\text{m ms}^{-1}$)	5.56 ±3.3	5.816 ±2.5
PGSE ($\kappa_{\text{sarco}} = 0\,\mu\text{m ms}^{-1}$)	1.82 ±1.3	1.2 ±1.3
PGSE ($\kappa_{\text{sarco}} = 0.05\,\mu\text{m ms}^{-1}$)	4.721 ±2.3	5.146 ±2.1
STEAM ($\kappa_{\text{sarco}} = 0\,\mu\text{m ms}^{-1}$)	1.827 ±0.9	1.324 ±1.2
STEAM ($\kappa_{\text{sarco}} = 0.05\,\mu\text{m ms}^{-1}$)	4.872 ±1.6	5.02 ±1.1

TABLE S4 Comparison of cones of uncertainty for MCSE, PGSE and STEAM sequences for the second and third eigenvectors across two distinct extreme permeabilities.

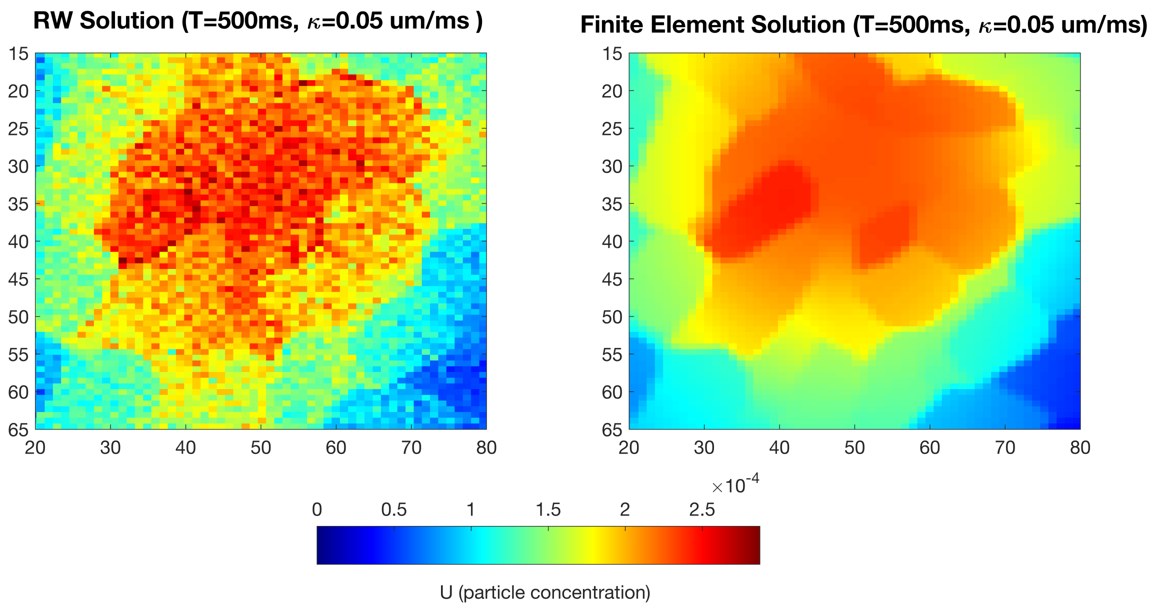

FIGURE S1 Comparison between Finite Element solution and Random Walk in a representative domain for $\kappa_{sarco} = 0.05 \mu m\, ms^{-1}$ and T=500ms. The FE solution was obtained considering Continuous Galerkin (CG) elements while assuming a small buffer region accounting for the membrane

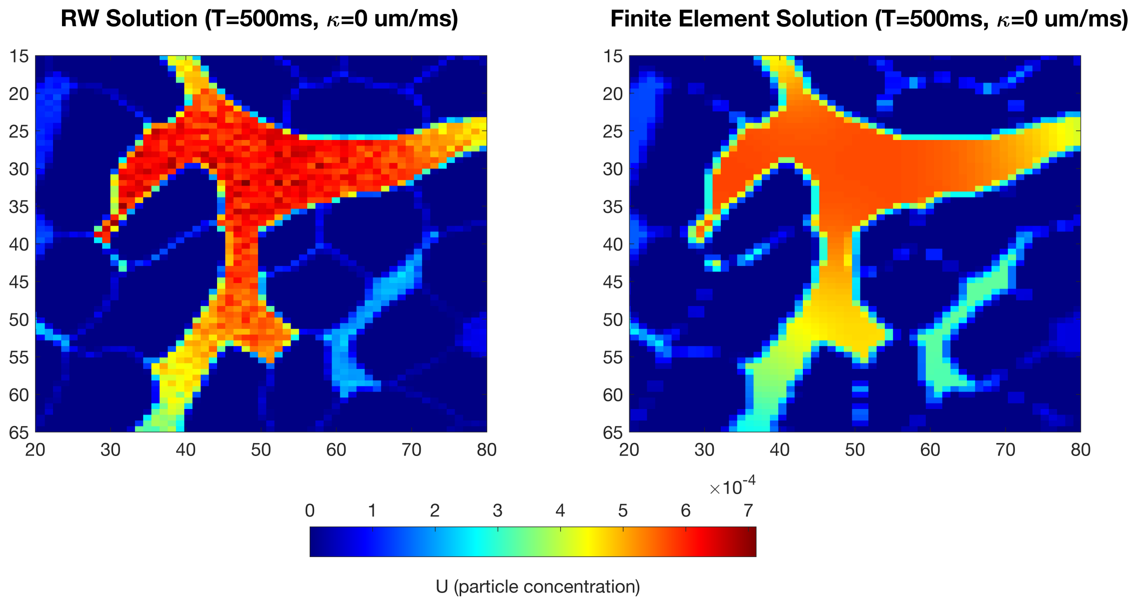

FIGURE S2 Comparison between Finite Element solution and Random Walk in a representative domain for $\kappa_{sarco} = 0 \mu m\, ms^{-1}$ and T=500ms. The FE solution was obtained considering Continuous Galerkin (CG) elements while assuming a small buffer region accounting for the membrane.

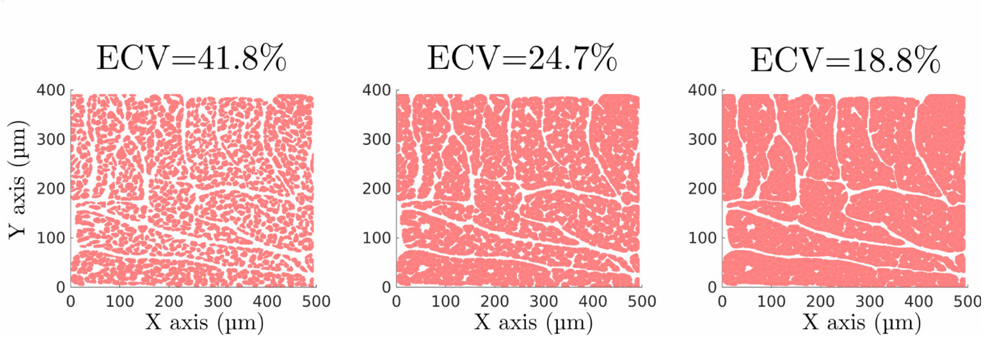

FIGURE S3 Illustration of three different extracellular volume fractions (ECV) obtained by morphing the manually segmented cardiomyocytes.

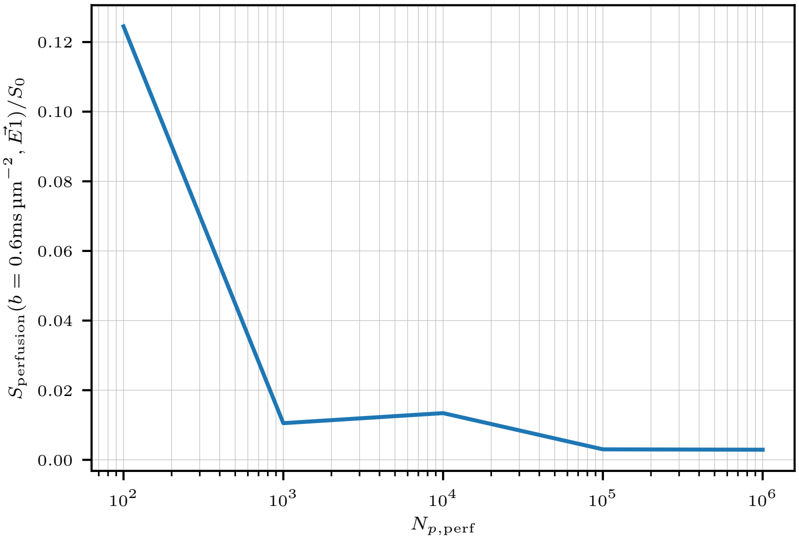

FIGURE S4 Convergence of the perfusion signal obtained for a b-value of 0.6 ms ms^{-2} for different number of perfusion particles $N_{p,\mathrm{perf}}$.

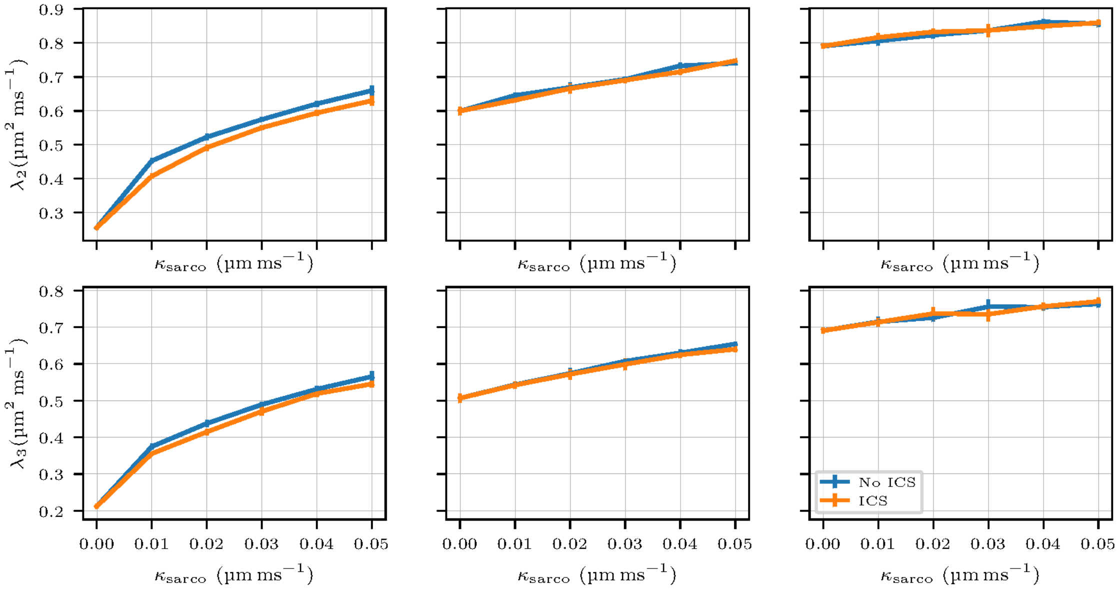

FIGURE S5 Illustration of the effect of ICD in three different sequences illustrating λ_2 and λ_3. For this plot, an ECV = 24.7% and a $\kappa_{\text{sarco}} = 0.05\,\mu\text{m}\,\text{ms}^{-1}$ have been considered. The different plots show mean values using 6 diffusion simulations with $N_{p,\text{diff}} = 10^5$ and $N_t = 10^4$ walkers with a 95% confidence interval. Note that we have considered $\kappa_{\text{ICD}} = 0.005\mu\text{m}\,\text{ms}^{-1}$ for all κ_{sarco} cases except for the impermeable case where $\kappa_{\text{ICD}} = \kappa_{\text{sarco}} = 0\mu\text{m}\,\text{ms}^{-1}$ has been considered.

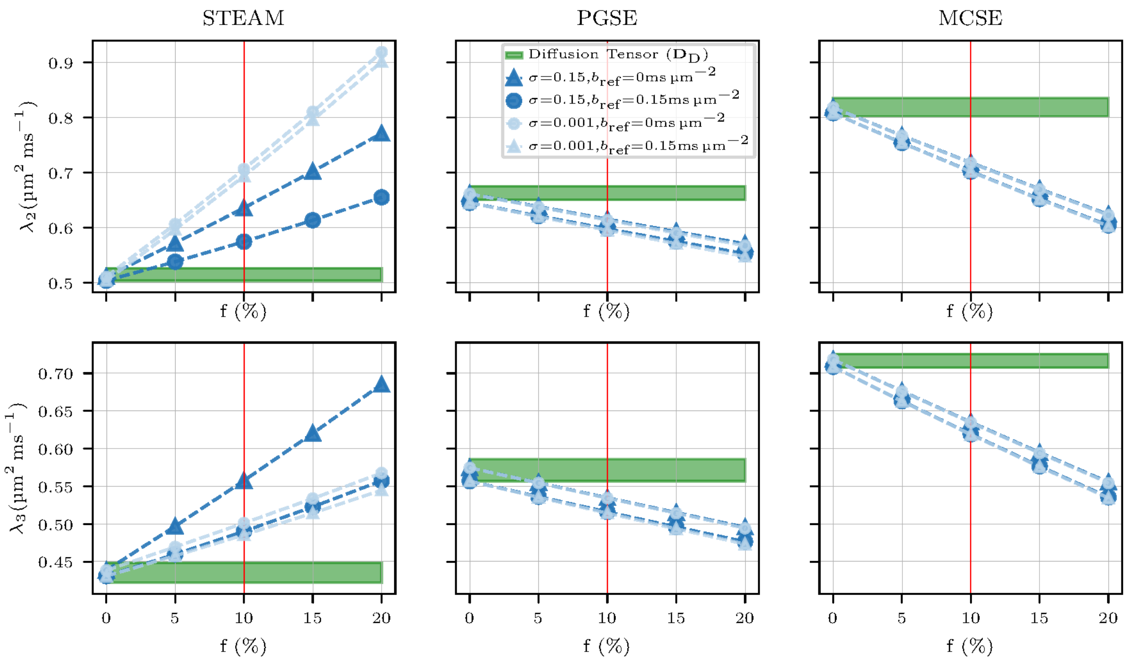

FIGURE S6 λ_2 and λ_3 parameters for several perfusion fraction values using a $K = 3.25$ (red vertical line representative of the capillary network in the myocardium). The green area shows the 95% confidence interval obtained in the diffusion simulations without the effect of perfusion. Two extreme inter-capillary velocities are plotted considering two distinct fittings tensor methods with b_{ref}=0 ms μm^{-2} and b_{ref}=0.15 ms μm^{-2}. The diffusion results have been obtained considering 6 simulations of 10^5 particles and 10^4 time steps while the perfusion simulations have been performed using only 1 simulation of 10^5 particles.

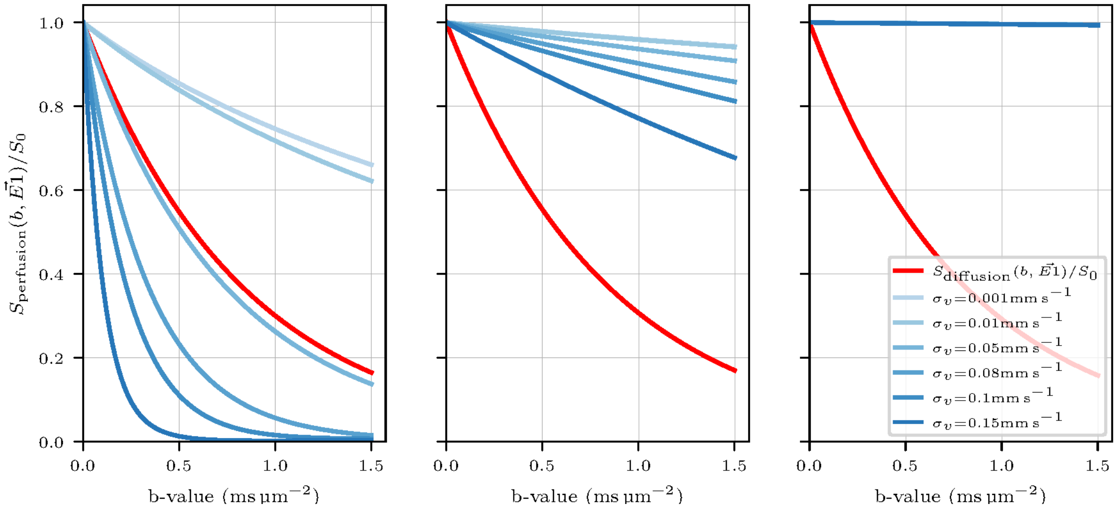

FIGURE S7 The perfusion and diffusion signal attenuations along $\vec{E}1$ are plotted for the three sequences considering several Gaussian inter-capillary velocities varying its standard deviation from σ_v = 0.001 mm s^{-1} to σ_v = 0.15 mm s^{-1}
